# Improving health in children with disabilities: an intervention-development study to support participation in leisure in 8-12 year olds with communication and mobility limitations

**DOI:** 10.1186/1745-6215-16-S2-P1

**Published:** 2015-11-16

**Authors:** Jennifer McAnuff, Allan Colver, Tim Rapley, Niina Kolehmainen

**Affiliations:** 1 Newcastle University, Newcastle upon Tyne, UK; 2 Leeds Community Healthcare NHS Trust, Leeds, UK

## 

Participation (i.e. involvement in life situations, such as leisure, education and self-care) is a fundamental health outcome for all children. Children with communication and mobility limitations experience restrictions to their participation in leisure, and the gap widens at age 8-12 years. These restrictions have negative consequences for their development and longer-term health and wellbeing. Evidence indicates that personal factors (e.g. child's motivation) and social-environmental factors (e.g. family behaviours) determine participation in leisure; however these pathways have rarely been systematically investigated and targeted in intervention-development research.

The proposed study will address three questions: What modifiable personal and social-environmental factors influence participation in leisure in 8-12 years with communication and mobility limitations? What intervention techniques should National Health Service (NHS) clinicians use to target these factors? And, how should clinicians go about delivering these techniques?

The methods will consist of two systematic reviews of current evidence; a Delphi study involving parents, clinicians and researchers; focus groups with parents, clinicians and children; and three mixed methods (QUANT+qual) single case studies.

The primary output will be an intervention manual containing: (i) clearly specified, replicable intervention techniques for targeting modifiable personal and social environmental factors related to children's participation in leisure; and (ii) clear descriptions of feasible and acceptable approaches to delivering the techniques in NHS settings. The manual will enable NHS clinicians to provide evidence-based participation support, and will be a basis for further evaluation of participation interventions techniques. The secondary output will be a design for a feasibility trial of selected intervention techniques.

